# Antifungal Susceptibility Patterns of *Candida* Species Recovered from Endotracheal Tube in an Intensive Care Unit

**DOI:** 10.1155/2016/9242031

**Published:** 2016-08-23

**Authors:** Elham Baghdadi, Sadegh Khodavaisy, Sassan Rezaie, Sara Abolghasem, Neda Kiasat, Zahra Salehi, Somayeh Sharifynia, Farzad Aala

**Affiliations:** ^1^Department of Microbiology, Faculty of Science, Islamic Azad University, Varamin-Pishva Branch, Tehran, Iran; ^2^Department of Medical Parasitology and Mycology, Kurdistan University of Medical Sciences, P.O. Box 14155-6446, Sanandaj, Iran; ^3^Division of Molecular Biology, Department of Medical Mycology and Parasitology, School of Public Health, Tehran University of Medical Sciences, Tehran, Iran; ^4^Department of Microbiology, Faculty of Science, Islamic Azad University, North Tehran Branch, Tehran, Iran; ^5^Department of Medical Mycology, Jondishapour University of Medical Sciences, Ahvaz, Iran; ^6^Department of Medical Mycology, Tarbiat Modares University, Tehran, Iran; ^7^Clinical Tuberculosis and Epidemiology Research Center, National Research Institute of Tuberculosis and Lung Diseases (NRITLD), Shahid Beheshti University of Medical Sciences, Tehran, Iran

## Abstract

*Aims*. Biofilms formed by* Candida* species which associated with drastically enhanced resistance against most antimicrobial agents. The aim of this study was to identify and determine the antifungal susceptibility pattern of* Candida* species isolated from endotracheal tubes from ICU patients.* Methods*. One hundred forty ICU patients with tracheal tubes who were intubated and mechanically ventilated were surveyed for endotracheal tube biofilms. Samples were processed for quantitative microbial culture. Yeast isolates were identified to the species level based on morphological characteristics and their identity was confirmed by PCR-RFLP. Antifungal susceptibility testing was determined according to CLSI document (M27-A3).* Results.* Ninety-five strains of* Candida* were obtained from endotracheal tubes of which* C. albicans* (*n* = 34; 35.7%) was the most frequently isolated species followed by other species which included* C. glabrata* (*n* = 24; 25.2%),* C. parapsilosis* (*n* = 16; 16.8%),* C. tropicalis* (*n* = 12; 12.6%), and* C. krusei* (*n* = 9; 9.4%). The resulting MIC_90_ for all* Candida* species were in increasing order as follows: caspofungin (0.5 *μ*g/mL); amphotericin B (2 *μ*g/mL); voriconazole (8.8 *μ*g/mL); itraconazole (16 *μ*g/mL); and fluconazole (64 *μ*g/mL).* Conclusion*.* Candida* species recovered from endotracheal tube are the most susceptible to caspofungin.

## 1. Introduction

Nosocomial infections are an important cause of mortality and increasing hospital costs [1]. The infection is usually transmitted to susceptible patients through infected medical staff and equipment by pathogenic organisms [2]. Biofilms are microbial communities embedded in biopolymer matrix on living or nonliving substrates [3]. According to the National Institutes of Health America, approximately 80% of hospital infections are associated with microbial biofilms [4]. Important microorganisms involved in microbial biofilm formation predominantly consist of many species of both fungal and bacterial. Among pathogenic fungi,* Candida* species are the most common cause of superficial and systemic disease [5].* Candida* species can aggregate on medical devices such as venous and urinary catheters, dentures, and ocular implants by biofilms [3, 6, 7].* Candida* biofilms can identify in individuals with certain circumstances, such as immunocompromised patients, HIV infected, cancerous, and organ transplant recipients. Ventilator-associated pneumonia is the most frequent intensive care unit (ICU) acquired infection among patients ventilated through tracheostomy or endotracheal intubation [8]. This topic can provide the conditions such as prolonged hospitalization and use of a variety of devices for colonization and creating biofilms by* Candida* species. Biofilms formed by these fungal organisms are associated with drastically enhanced resistance against most antifungal therapy [8, 9]. Several studies show that antifungal agents, that is, amphotericin B, fluconazole, itraconazole, and ketoconazole, displayed less activity against* Candida albicans* biofilms formed on the PVC disk [10–12]. In addition, non-*C. albicans* have shown resistance to two new antifungal drugs (voriconazole and ravuconazole), but it seems that antibiofilm activity of amphotericin B lipid formulation and also echinocandins have existed [13]. Therefore the aim of the present study was to evaluate antifungal susceptibility testing of* Candida* spp. isolated from endotracheal tubes in ICU patients.

## 2. Materials and Methods

This study was carried out in the Intensive Care Unit of the Imam Khomeini and Golestan Hospitals, Ahvaz, during January–September 2015. One hundred forty ICU patients with tracheal tubes who were intubated and mechanically ventilated were surveyed for endotracheal tube biofilms. The length of hospitalization was at least two weeks prior to sampling. Collected endotracheal tubes of patients who had clinical manifestation of pneumonia including cough, purulent respiratory secretion, fever, and new or progressive infiltration of lung in CXR were placed in sealed sterile bottles and referred immediately to the laboratory for processing. From the central region of each endotracheal tube 1 cm section was cut and processed for quantitative microbial culture.

### 2.1. Morphological and Molecular Identification

The specimens were inoculated onto Sabouraud Dextrose Agar (SDA, Difco) supplemented with chloramphenicol and incubated at 37°C for two days. Primarily, yeast colonies were identified by conventional tools such as colony color on CHROMagar* Candida* medium (CHROMagar Company, Paris, France), germ tube tests in serum at 37°C for 2-3 h, and microscopic morphology on cornmeal agar (Difco Laboratories, Detroit, Mich., USA) with 1% Tween 80. Confirmation molecular approaches were adjusted. Genomic DNA was extracted, using the method of glass bead disruption, and the PCR-RFLP method was performed as described previously [14].

### 2.2. Antifungal Susceptibility Testing

MICs (minimum inhibitory concentrations) of identified* Candida* isolates were determined according to recommendations stated in the Clinical and Laboratory Standards Institute (CLSI) M27-A3 document [15]. Amphotericin B (Sigma, St. Louis, MO, USA), fluconazole (Pfizer, Groton, CT, USA), itraconazole (Janssen Research Foundation, Beerse, Belgium), voriconazole (Pfizer, Groton, CT, USA), and caspofungin (Merck, Whitehouse Station, NJ, USA) were obtained as reagent-grade powders from the respective manufacturers for preparation of the CLSI microdilution trays. Inoculum was prepared by gently scraping the surface of the fungal colonies with a sterile cotton swab moistened with sterile physiological saline. Conidial suspensions were adjusted to transmission of 75% to 77% at 530 nm (approximate 1 × 10^6^–5 × 10^6^ CFU/mL). The inoculum suspensions, including mostly nongerminated conidia, were diluted 1 : 1000 in RPMI 1640 medium and the final inoculum in assay wells was between 0.5 × 10^3^ and 5 × 10^3^ CFU/mL. The microdilution trays were incubated at 35°C for 24–48 h. MICs were determined visually by comparison of the growth in the wells containing the drug with the drug-free control.* Candida parapsilosis* (ATCC 22019) and* Candida krusei* (ATCC 6258) reference strains were for quality control.

## 3. Results

Out of one hundred forty ICU patients hospitalized, ninety-five strains of* Candida* which were obtained from endotracheal tubes were studied. The positive specimens belonged to 67 male and 28 female hospitalized patients. The duration of being intubated had a median of 9 days and hospital stay duration average was 29 ± 3.6 days. The isolates were confirmed based on species level using PCR-RFLP of which* C. albicans* (*n* = 34; 35.7%) was the most frequently isolated species, followed by* C. glabrata* (*n* = 24; 25.2%),* C. parapsilosis* (*n* = 16; 16.8%),* C. tropicalis* (*n* = 12; 12.6%), and* C. krusei* (*n* = 9; 9.4%).  Table 1 summarizes the results of* in vitro* antifungal susceptibility profiles (MIC range, geometric mean MIC, MIC_50_, and MIC_90_) of several antifungal drugs against all* Candida* species. The resulting MIC_50_ for all* Candida* species were in increasing order as follows: caspofungin (0.5 *μ*g/mL); amphotericin B (1 *μ*g/mL); voriconazole (0.25 *μ*g/mL); itraconazole (0.75 *μ*g/mL); and fluconazole (4 *μ*g/mL). Results showed the widest range and the highest MICs for fluconazole (0.016–≥64 *μ*g/mL), voriconazole (0.016–≥16 *μ*g/mL), and itraconazole (0.016–≥16 *μ*g/mL). Results revealed statistically significant differences when comparing the susceptibility of* C. albicans* and non-*albicans Candida* to fluconazole, voriconazole, and itraconazole (*P* < 0.05) with* C. albicans* being found to be the most susceptible to these antifungal agents. MIC results among all the isolates of* Candida* species showed that they were fully susceptible to caspofungin (Table 2).

## 4. Discussion

*Candida* is opportunistic pathogen which causes a life-threatening infection with high rates of mortality especially in immunocompromised individuals [16]. The pathogenicity of* Candida* species is attributed to certain virulence factors, mostly production of biofilm [17, 18].* Candida* species are now recognized as major agents of hospital-acquired infection. Almost invariably, an implanted device such as an urinary catheter or endotracheal tube is associated with these infections [18].* Candida* species can cause significant problems of medical settings as persistent and recurrent device related infections [19, 20]. This properties also differed among different species of* Candida* [17, 21]. In this study* C. albicans* (35.7%) was the most common species obtained from endotracheal tube, compatible with other studies that mentioned that* C. albicans* is considered as major etiologic agent in candidiasis [16, 17, 20]. Other studies reported that the ability of biofilm production in* C. parapsilosis* and* C. glabrata* was significantly less than* C. albicans* [17, 21]. Biofilm phenotype in non-*C. albicans* species is the cause of the survival and well adapted to colonization of tissues and indwelling devices [20]. This difference in our results probably is due to variety of biofilms formation among* Candida* species from different sources. Mahmoudabadi et al. showed a higher percentage* C. albicans* (41.7%) which have recovered from blood samples in comparison with other sources [21]. In our investigation, other species of* Candida* included* C. glabrata* (25.2%),* C. parapsilosis* (16.8%),* C. tropicalis* (12.6%), and* C. krusei* (9.4%) obtained from endotracheal tubes. These data are in agreement with the findings of a previous study [22–25]. Also Shokohi et al., Richter et al., and Papon et al. mentioned* C. glabrata* as the most common non*-C. albicans* species in their investigation [24–27]. Deorukhkar et al.'s study indicated* C. tropicalis* (29.4%) as the major non-*C. albicans* species isolate followed by* C. glabrata* (20.7%) that is incompatible with these studies [28, 29]. The obtained antifungal susceptibility patterns indicated that* C. albicans* isolates were highly susceptible to caspofungin (100%) (MIC ≤ 2 *μ*g/mL). These findings are in agreements with other studies that reported [30, 31]. In this investigation 26.4% of* C. albicans* strains were resistant to fluconazole (MIC ≥ 64 *μ*g/mL), whereas other studies reported the rates of this resistance as 45.83%, 11.9%, 74.2%, 2.7%, and 38.7%, respectively [27, 32–35]. Studies by Shokohi et al., Al-Mamari et al., Aher et al., Awari, and Roy et al. indicated the resistance of* C. albicans* to itraconazole as 5.4%, 10.3%, 36.9%, 35%, and 19.3%, respectively [26, 32, 36, 37]. However, in our finding 35.2% of* C. albicans* strains were shown to be resistant to itraconazole MIC ≥ 1 *μ*g/mL. In our study 14.7% of* C. albicans* isolates were resistant to voriconazole (MIC ≥ 1 *μ*g/mL) that was different from results of Zang et al. and Badiee and Alborzi's studies [23, 38]. MIC range (0.016–≥16 *μ*g/mL) and MIC_90_ (8 *μ*g/mL) for voriconazole in present study were different from results by Zhang et al. and Badiee and Alborzi which reported MIC range and MIC90 as 0.0313–4 *μ*g/mL and 0.25 *μ*g/mL and 0.003–16 *μ*g/mL and 4 *μ*g/mL, respectively [23, 38]. In addition, 17.6% of* C. albicans* isolates in our study were indicated to be resistant to amphotericin B MIC ≥ 2 *μ*g/mL. The result was to some extent similar to the result reported by of Aher et al. (13.8%) and differs from results by Njunda et al. (54.4%), Awari et al. (7.5%), and Zhang et al. (1.1%) [26, 33, 35, 37–39]. MIC range (0.062–2 *μ*g/mL) and MIC_90_ (2 *μ*g/mL) for amphotericin B in present study differ from the data reported by Bosco-Borgeat et al. which reported MIC Range and MIC_90_ as 0.13–1 *μ*g/mL and 0.5 *μ*g/mL, respectively [39]. Our data indicated that 79.1% of* C. glabrata *were resistance to itraconazole. These data are in disagreement with the rate of itraconazole resistance* C. glabrata *in studies by Shokohi et al. (12.5%), Haddadi et al. (21%), and Deorukhkar et al. (46.2%) [27, 29, 40]. Also Aher reported 46.4% and 40% resistance to fluconazole and itraconazole, respectively; these mentioned rates differ from the results of our investigation [26]. Our study has shown that fluconazole MIC_90_ values (8, 64, and 2 *μ*g/mL), itraconazole (16, 16, and 0.5 *μ*g/mL), and voriconazole (8, 16, and 0.25) against* C. albicans, C. glabrata*, and* C. parapsilosis,* respectively, according to study by Badiee and Alborzi in regard to fluconazole rates are lower (16, 128, and 4) and itraconazole and voriconazole have higher amount (2, 16, and 0.25 and 4, 3, and 0.033), respectively [23]. In fact, MIC_90_ in* C. glabrata* as the main non*-Candida albicans* to fluconazole, itraconazole, and voriconazole was higher than* C. albicans* and* C. parapsilosis*. MIC_90_ fluconazole, itraconazole, and voriconazole for* C. glabrata *in our investigation were 64, 16, and 16. Long term fluconazole and itraconazole prophylaxis were accompanied with reduction in sensitivity to these agents and recently* C. glabrata* known as naturally less susceptible to azoles compared to other* Candida* species [35, 41, 42]. Our study has shown that amphotericin B and caspofungin MIC_90_ values were 2, 2, and 0.5 *μ*g/mL and 0.5, 0.75, and 0.5 *μ*g/mL against* C. albicans, C. glabrata*, and* C. parapsilosis,* respectively. However in other studies MIC_90_ values of amphotericin B were lower (0.25, 0.5, and 0.25 *μ*g/mL) [23]. In present study 87.5%, 37.5%, and 75% of* C. parapsilosis* strains were susceptible to fluconazole, itraconazole, and voriconazole, respectively. This result is similar to that by Shokohi et al. who found no resistance species among them. Badiee et al. obtained 6.9% and 3.5% resistance to fluconazole and itraconazole, respectively. In addition they find no voriconazole resistance* C. parapsilosis* among them. In addition Zhang et al.'s findings show 15.4% resistance to fluconazole; however there was no resistance to itraconazol and voriconazole [23, 27, 38]. In our study we find that 41.6%, 66.6%, and 41.6% of* C. tropicalis *were resistant to fluconazole, itraconazole, and voriconazole, respectively. In contrast, Shokohi et al. did not find resistance species in their study. Also Zhang et al. and Aher et al. obtained 10.7% and 4.8% and 52% and 56% of isolates which were resistant to fluconazole and itraconazole, respectively [26, 27, 38]. However* C. tropicalis* isolates were highly susceptible to caspofungin and amphotericin B (100%, 83.3%). Therefore these antifungals seem to be the most active drug for candidiasis treatment. In our study MIC_90_ of fluconazole, itraconazole, and voriconazole for* C. parapsilosis* were 2, 0.5, and 0.25 *μ*g/mL whereas Zhang et al., Tay et al., and Bonfietti et al. obtained 2, 0.062, and 0.25 *μ*g/mL, 4, 0.19, and 0.047 *μ*g/mL, 2, 0.06, and 0.03 *μ*g/mL, respectively, as MIC_90_ to mention antifungal drugs [38, 43, 44]. Our results indicated that* C. parapsilosis* isolates from endotracheal tube were highly susceptible to caspofungin and amphotericin B; this data also shows that the concentration of 0.5 *μ*g/mL of this medicine is able to inhibit 90% of mentioned isolates.

## 5. Conclusion

Knowledge of* Candida* species distribution and antifungal resistance pattern of them plays an important role in appropriate therapy. Our results suggest that* Candida* species recovered from endotracheal tube are the most susceptible to caspofungin, followed by amphotericin B, voriconazole, itraconazole, and fluconazole.

## Figures and Tables

**Table 1:** *In vitro *susceptibilities of *Candida* spp. recovered from endotracheal tube to antifungal agents. MIC range, geometric mean, MIC_50_, and MIC_90_ values are expressed in *μ*g/mL.

Species (*n*)	Antifungal agents	Ranges	MIC_50_	MIC_90_	GM
Total* Candida *spp. (95)	Amphotericin B	0.062–4	1	2	0.420
	Itraconazole	0.016–≥16	0.75	4	0.847
	Voriconazole	0.016–≥16	0.25	4	0.381
	Fluconazole	0.016–≥64	4	64	2.881
	Caspofungin	0.008–2	0.5	0.5	0.294

*C. albicans* (34)	Amphotericin B	0.062–2	0.5	2	0.178
	Itraconazole	0.016–≥16	0.062	16	0.242
	Voriconazole	0.016–≥16	0.031	8	0.119
	Fluconazole	0.016–≥64	0.5	8	0.999
	Caspofungin	0.008–0.5	0.5	0.5	0.182

*C. glabrata* (24)	Amphotericin B	0.062–2	1	2	0.706
	Itraconazole	0.016–16	8	16	3.121
	Voriconazole	0.016–16	0.5	16	0.684
	Fluconazole	0.25–16	16	64	5.941
	Caspofungin	0.125–1	0.5	0.75	0.390

*C. parapsilosis* (16)	Amphotericin B	0.016–1	0.25	0.5	0.158
	Itraconazole	0.016–0.5	0.5	0.5	0.249
	Voriconazole	0.016–0.5	0.125	0.25	0.157
	Fluconazole	0.125–4	2	2	1
	Caspofungin	0.125–0.5	0.5	0.5	0.314

*C. tropicalis* (12)	Amphotericin B	1–4	2	4	2
	Itraconazole	1–≥64	16	16	7.245
	Voriconazole	0.125–4	2	2	1
	Fluconazole	4–≥64	16	—	19.02
	Caspofungin	0.008–2	0.5	0.5	0.458

*C. krusei* (9)	Amphotericin B	0.062–2	2	—	0.447
	Itraconazole	0.031–8	0.37	—	0.398
	Voriconazole	0.125–16	0.25	—	0.870
	Fluconazole	0.016–4	32	—	2.512
	Caspofungin	0.008–2	0.375	—	0.353

GM: geometric mean.

**Table 2:** MIC interpretation of five antifungal drugs against *Candida* spp. recovered from endotracheal tube.

Antifungal agents		*C. albicans* *n* = 34 (%)	*C. glabrata* *n* = 24 (%)	*C. parapsilosis* *n* = 16 (%)	*C. tropicalis* *n* = 12 (%)	*C. krusei* *n* = 9 (%)
Amphotericin B	S	28 (82.3)	20 (83.3)	16 (100)	10 (83.3)	7 (77.7)
	R	6 (17.6)	4 (16.6)	—	2 (16.7)	2 (22.2)

Itraconazole	S	20 (58.8)	12 (50)	6 (37.5)	4 (33.3)	3 (33.3)
	DD	2 (5.8)	6 (25)	10 (62.5)	—	5 (55.5)
	R	12 (35.2)	6 (25)	—	8 (66.6)	1 (11.1)

Voriconazole	S	27 (79.4)	11 (45.8)	12 (75)	4 (33.3)	6 (66.6)
	DD	2 (5.8)	5 (20.8)	4 (25)	3 (25)	2 (22.2)
	R	5 (14.7)	8 (33.3)	—	5 (41.6)	1 (11.1)

Fluconazole	S	22 (64.7)	3 (12.5)	14 (87.5)	3 (25)	5 (55.5)
	DD	3 (8.8)	2 (8.3)	2 (12.5)	4 (33.3)	2 (22.2)
	R	9 (26.4)	19 (79.1)	—	5 (41.6)	2 (22.2)

Caspofungin	S	34 (100)	24 (100)	16 (100)	12 (100)	9 (100)
	R	—	—	—	—	—

S: susceptible; R: resistance; DD: dose-dependent.
